# A Survey on Error Exponents in Distributed Hypothesis Testing: Connections with Information Theory, Interpretations, and Applications

**DOI:** 10.3390/e26070596

**Published:** 2024-07-12

**Authors:** Sebastián Espinosa, Jorge F. Silva, Sandra Céspedes

**Affiliations:** 1 Department of Electrical Engineering, Universidad de Chile, Santiago 9170022, Chile; sebastian.espinosa@ing.uchile.cl; 2 Department of Computer Science & Software Engineering, Concordia University, Montreal, QC H3G 1M8, Canada; sandra.cespedes@concordia.ca

**Keywords:** hypothesis testing, distributed inference, sensor networks, performance bounds, finite-length analysis, error exponent, mutual information, information bottleneck

## Abstract

A central challenge in hypothesis testing (HT) lies in determining the optimal balance between Type I (false positive) and Type II (non-detection or false negative) error probabilities. Analyzing these errors’ exponential rate of convergence, known as error exponents, provides crucial insights into system performance. Error exponents offer a lens through which we can understand how operational restrictions, such as resource constraints and impairments in communications, affect the accuracy of distributed inference in networked systems. This survey presents a comprehensive review of key results in HT, from the foundational *Stein’s Lemma* to recent advancements in distributed HT, all unified through the framework of error exponents. We explore asymptotic and non-asymptotic results, highlighting their implications for designing robust and efficient networked systems, such as event detection through lossy wireless sensor monitoring networks, collective perception-based object detection in vehicular environments, and clock synchronization in distributed environments, among others. We show that understanding the role of error exponents provides a valuable tool for optimizing decision-making and improving the reliability of networked systems.

## 1. Introduction

Hypothesis testing (HT) is a fundamental problem in statistics and information theory, playing a crucial role in decision-making, digital communication, quality control, medical diagnosis, and more [1,2,3,4,5]. HT makes inferences based on observations and provides a structured framework for evaluating claims or hypotheses against observed evidence. At the heart of hypothesis testing is the need to balance two types of errors: Type I (false positive) and Type II (non-detection or false negative). The probabilities of these errors assess the reliability and quality of a test.

Error exponents quantify in a setting of multiple observations the feasible rate at which the probabilities of the mentioned errors decrease as the size of the observation (or sample size) increases [1,6,7,8]. They offer an elegant method to quantify the complexity and difficulty of a given testing scenario. Understanding error exponents can guide researchers and practitioners in selecting appropriate testing procedures and designing systems with the desired level of accuracy [9,10,11].

In this survey, we explore hypothesis testing (HT) from its error exponents characterization, focusing on applications in distributed sensor networks that employ three different HT strategies: centralized, distributed, and collaborative. We aim to highlight the relevant role of error exponents in the asymptotic and non-asymptotic analysis of the error probabilities in HT. Our study provides insights into designing robust hypothesis testing systems, enhancing the accuracy of decision-making processes, and understanding the trade-offs involved in minimizing error probabilities. Through this exploration, we seek to advance the understanding and use of HT in various fields, contributing to a more profound knowledge of its implications in real-world scenarios.

### 1.1. Centralized HT

The classical setting in statistics involves making decisions based on data collected from a single source or location. In this framework, the hypotheses are traditionally formulated regarding the parameters or characteristics of the probability law of a population or system. The test uses data from a lossless observation (sample) to either accept or reject the null hypothesis. The methods often compare the evidence (sample) with theoretical distributions under the null and the alternative hypotheses to assess the likelihood of observing the data. In this context, classical results regarding the optimality of a test and statistical properties for various tests are well established [1,12]. These classical results are the foundation for HT in centralized scenarios, offering insights into the performance and characteristics of different testing procedures.

### 1.2. Distributed HT

The mentioned landscape changes dramatically in a distributed scenario. Unlike centralized HT, distributed HT involves multiple agents or sensors that collect and process data in a distributed way before communicating information to a central decision-maker. The problem of distributed inference is fundamental from a theoretical perspective [13,14,15] and relevant in numerous sensor networks and Internet of Things (IoT) applications [16,17,18,19,20,21,22]. As an important motivation, the field of the Internet of Things (IoT) adds new dimensions and technical difficulties to traditional decision-making problems, as data are no longer centrally accessible for inference. Then, we must address several technical issues related to data transmission, sensor noise, adversarial disruptions, and unordered data. These challenges are highlighted in works, such as [23,24,25,26,27,28,29,30]. In this scenario, a crucial problem in network inference is the task of a distributed decision under communication constraints [14,28,29,30].

Distributed detection, data fusion, and multisensor integration have deep roots in statistics, signal processing, and communication. Foundational contributions can be traced to works, such as [31,32,33]. Decentralized decision frameworks have applications in communications and sensor networks, for example, in Multiple Access Channels (MACs) [34] and wireless sensor networks [16]. A case of interest is a fusion center obtaining quantized measurements from remote sensors [35,36]. In this setting, many works have studied the asymptotic performance limits in distributed scenarios with multiple decision centers and rate constraints between sensors and detectors [37,38,39]. Other notable applications include spectrum sensing in cognitive radio networks, where a primary agent and a group of secondary agents collaborate to detect the presence of the primary user’s signal, introducing dependence among the decentralized sensors [19,20]. Additionally, other applications have been developed in the contexts of censoring and security, such as detecting the independence of two remotely located sources in the presence of an eavesdropper [21,22].

This decentralized setup presents new challenges and complexities, requiring the development of innovative methodologies and frameworks to address issues, such as communication constraints, coordination among agents, and optimal decision-making in distributed environments [24,25,26,27,28,29,30,37,38,39]. Importantly, classical results from (centralized) HT cannot be directly applied in distributed HT settings. New approaches tailored to distributed settings are required to derive fundamental performance limits (error exponents) and propose optimal HT schemes. In distributed HT, there are multiple communication configurations among sensors. Therefore, the characterization of the error exponent will vary depending on the configuration, and there may not necessarily be optimality guarantees for each scenario, as will be covered in some detail in this survey.

### 1.3. The Seminal One-Sided Distributed Result

A notable development in distributed HT is the one-sided distributed setting introduced by Ahlswede and Csiszár in their paper [14]. This problem is a test against independence using observations from two remote nodes. Specifically, a sensor node transmits to another remote agent (the detector) under a rate constraint in bits per sample. In this context, [14] conducted an information-theoretic analysis of the one-sided distributed HT problem, deriving the asymptotic (error exponent) limit for the Type II error under a fixed Type I error constraint. Espinosa et al. recently presented finite-length performance bounds for this one-sided distributed problem [15,40,41]. These non-asymptotic bounds are essential because they inform the test designer what can be achieved with an optimal encoder and detector when a finite number of observations are available for decision-making. Furthermore, these performance bounds show how quickly the error exponents are reached as the number of samples grows. These results offer insight into how well the error exponents represent the performance of real-world decision schemes that operate with limited observations [15].

### 1.4. Scope, Content, and Organization

The necessity of conducting this survey on distributed HT arises from the fragmented nature of the current literature in this field that might not be easily accessible to the broader community interested in HT. To our knowledge, no existing study unifies and consolidates findings from centralized to distributed HT, focusing on an error exponent analysis. Our work significantly contributes to this endeavor by coherently presenting a progression of relevant results and their interpretations. This survey aims to facilitate a better understanding of the existing results of error exponents to a broader community interested in HT applications in distributed settings.

In the forthcoming sections, we delve into the intricacies of error exponent analysis in HT, beginning with some classical results (centralized within this survey) and continuing with this survey’s primary focus on distributed HT results. Furthermore, we will explore the established findings in both asymptotic and finite-length analyses, elucidating the significance of each. This endeavor encompasses six pivotal axes, each of which will be succinctly delineated in the ensuing discourse. The six axes of this survey can be summarized as follows:

- Centralized HT: This approach involves a central authority or entity that receives and processes data to make decisions. Centralized testing is standard in traditional statistical applications, where a single decision-maker evaluates evidence and makes conclusions.
- Distributed HT: Multiple entities share information in a distributed setup to make collective decisions. This approach is essential in networks of sensor systems and collaborative environments where no single entity can access the complete (lossless) data.
- Collaborative HT: This is a special case of distributed HT. The entities communicate and retransmit their local information between them to make a collective decision about the hypothesis.
- Asymptotic analysis: This axis entails studying the behavior of error probabilities as the data size tends toward infinity. This approach allows researchers to examine the fundamental limits of statistical inference, particularly in scenarios involving large sample sizes. In information theory, asymptotic analysis is crucial in understanding the trade-off between error rates and the amount of information transmitted (in bits per sample). This analysis helps establish theoretical benchmarks and informs the practitioner in designing communication systems and statistical inference methods that operate efficiently.
- Finite-length analysis: While the error exponent analysis of HT relies on a large sampling regime, the non-asymptotic analysis addresses scenarios with finite data and a smaller sample size. Therefore, a finite-length analysis considers the practical constraints of real-world scenarios where resources are constrained. Using non-asymptotic results, researchers can assess the trade-offs between performance, computational complexity, and resource utilization, thereby facilitating the development of efficient and reliable systems for processing and transmission in real-world applications.
- Applications: Discussing practical applications of error exponents in communications is crucial because it showcases their concrete uses and demonstrates how they can enhance performance. By exploring these applications, we can understand how error exponents make communication systems more efficient. This section will provide valuable insights into how these theoretical tools can effectively apply to real-world scenarios, highlighting their importance in modern communication technologies.

The rest of this survey is organized as follows. Section 2 will focus on centralized HT, discussing the fundamental principles and methodologies. The non-asymptotic counterpart of centralized HT is analyzed in Section 3. Section 4 will cover distributed HT, exploring different schemes. The particular case of the one-sided HT test is analyzed in Section 5, and both the asymptotic and non-asymptotic analyses of the test against independence are relegated to Section 6. Section 7 will examine interactive HT, a strategy that allows for nodes to share information and update their decisions based on the exchanged data, and, finally, in Section 8, we discuss the applications of error exponents in communications, distributed inference, sensor networks, and other areas.

### 1.5. Basic Notation and Conventions

Upper-case letters are used for random variables, and lower-case letters are used to represent realizations of random variables. Vectors are denoted by $X_{1}^{n}=\left(X_{1},\ldots ,X_{n}\right)$ with their length as superscripts. In this survey, all random variables are defined in finite alphabets. $P_{X}\in P\left(X\right)$ denotes the distribution for a random variable *X* defined on the set X, and P(X) is the set of distributions over X. The notation X ―○ Y ―○ Z indicates that *X*, *Y*, and *Z* form a Markov chain. Let ${\left(b_{n}\right)}_{n\geq 1}$ and ${\left(a_{n}\right)}_{n\geq 1}$ be sequences, $\left(b_{n}\right)=o\left(a_{n}\right)$ indicates that ${lim sup}_{n\to \infty }\left(b_{n}/a_{n}\right)=0$, and $\left(b_{n}\right)=𝒪\left(a_{n}\right)$ indicates that ${lim sup}_{n\to \infty }\left|b_{n}/a_{n}\right|<\infty$. We say that $\left(a_{n}\right)\approx \left(b_{n}\right)$ if for a sufficiently large N>0, there is a constant C>0 such that $a_{n}=Cb_{n}$, for all n≥N.

## 2. Centralized Hypothesis Testing (HT)

Binary hypothesis testing (BHT) is a standard centralized decision task that has been richly used in applications as diverse as signal detection [16,17], digital communication, anomaly detection, astronomical inference, pattern recognition, and model drift detection, to name a few examples [1,2,3,4,5].

Let us consider the classical *n*-length BHT setting given by

(1) $$\left\{\begin{array}{cc} H_{0}: & \;X_{1}^{n}∼P^{n}\\ H_{1}: & \;X_{1}^{n}∼Q^{n} \end{array}\right.$$

where $X_{1}^{n}$ represents the evidence (observation). The observation $X_{1}^{n}$ corresponds to *n* independent and identically distributed (i.i.d.) realizations of a random variable *X* that follows either *P* (under $H_{0}$) or *Q* (under $H_{1}$), where *P* and *Q* are probability distributions in P(X). In this exposition, we restrict our attention to the case where *X* takes values in a finite-alphabet space X, and P(X) is the family of probabilities on X.

In this context, a decision rule $\phi_{n}\left(\cdot \right)$ of length *n* is a function $\phi_{n}:X^{n}\to \Theta =\left\{0,1\right\},$ from which the two types of errors of BHT can be introduced [42]:

(2) $$\begin{array}{cc} P_{0}\left(\phi_{n}\right) & \equiv P^{n}\left(\left\{x_{1}^{n}\in X^{n}:\phi_{n}\left(x_{1}^{n}\right)\neq 0\right\}\right)=P^{n}\left(A^{c}\left(\phi_{n}\right)\right), \end{array}$$

(3) $$\begin{array}{cc} P_{1}\left(\phi_{n}\right) & \equiv Q^{n}\left(\left\{x_{1}^{n}\in X^{n}:\phi_{n}\left(x_{1}^{n}\right)=0\right\}\right)=Q^{n}\left(A\left(\phi_{n}\right)\right), \end{array}$$

where $A\left(\phi_{n}\right)\equiv \left\{x_{1}^{n}\in X^{n}:\phi_{n}\left(x_{1}^{n}\right)=0\right\}$.

### 2.1. The Neyman–Pearson Lemma

For a given ϵ>0, let us consider

(4) $$\beta_{n}\left(\epsilon \right)\equiv \min_{\begin{array}{c} \phi_{n}\in \Phi_{n} \end{array}}\left\{P_{1}\left(\phi_{n}\right):\;s.t.\;P_{0}\left(\phi_{n}\right)\leq \epsilon \right\},\forall n\geq 1,$$

where $\Phi_{n}\equiv \left\{\phi_{n}:X^{n}\to \Theta \right\}$ represents the complete class of *n*-length detectors. $\beta_{n}\left(\epsilon \right)$ is the best Type II error that can be achieved given a restriction (or fidelity requirement) on the Type I error. The well-known *Neyman–Pearson lemma* [32,43] characterizes a feasible solution for Equation (4). In particular, this lemma states that a solution of Equation (4) admits the following structure

(5) $$\phi_{n}^{\tau }\left(x_{1}^{n}\right)\equiv \left\{\begin{array}{cc} 1 & \;\mathrm{if}\;P^{n}\left(\left\{x_{1}^{n}\right\}\right)>\tau Q^{n}\left(\left\{x_{1}^{n}\right\}\right)\\ 0 & \;\mathrm{if}\;P^{n}\left(\left\{x_{1}^{n}\right\}\right)\leq \tau Q^{n}\left(\left\{x_{1}^{n}\right\}\right) \end{array}\right.$$

where $\tau \in R^{+}$. This family of detectors offers the optimal trade-off between the two types of errors [1,43].

If we consider a sequence ${\left(\epsilon_{n}\right)}_{n\geq 1}$ of non-negative values such that $\lim_{n\to \infty }\epsilon_{n}=0$, the sequence ${\left(\beta_{n}\left(\epsilon_{n}\right)\right)}_{n\geq 1}$ represents the optimum Type II error dynamics that satisfy a family of fixed Type I error constraints that vanish with the length of the observation vector. An important question that this survey will study is the characterization of the convergence dynamics (rate of convergence) of ${\left(\beta_{n}\left(\epsilon_{n}\right)\right)}_{n\geq 1}$ under various settings and conditions. Specifically, at what rate does the Type II error decrease to zero? How does this rate depend on properties of ${\left(\epsilon_{n}\right)}_{n\geq 1}$ and the two distributions *P* and *Q* introduced in (1)? The following subsections will address these questions, describing the asymptotic and non-asymptotic results.

### 2.2. Asymptotic Analysis and Error Exponent

A central question in information theory [1,7,44,45,46,47] has been determining the exact (exponential) rate of convergence of ${\left(\beta_{n}\left(\epsilon_{n}\right)\right)}_{n}$ (the Type II error) known as the error exponent of an HT task. The error exponent has been interpreted as an indicator of the complexity of a decision task, which is a function of *P*, *Q*, and ${\left(\epsilon_{n}\right)}_{n}$ of the presented setting.

For the simple case when $\epsilon_{n}=\epsilon >0$ for all *n*, i.e., there is a fixed Type I restriction, the celebrated *Stein’s lemma* determines that the error exponent of the Type II error is given by the *Kullback–Leibler divergence* (KLD) of *P* with respect to *Q* given by [1,42]

(6) $$D\left(P∥Q\right)\equiv \sum_{x\in X}P\left(\left\{x\right\}\right)\log\frac{P(\{x\})}{Q(\{x\})}.$$

**Lemma** **1** (*Stein’s lemma* [1,48])**.** *For any value of ϵ∈(0,1), the solutions of* (4) *satisfy that:*

(7) $$\lim_{n\to \infty }-\frac{1}{n}\log\left(\beta_{n}\left(\epsilon \right)\right)=D\left(P∥Q\right).$$

This result shows that ${\left(\beta_{n}\left(\epsilon_{n}\right)\right)}_{n\geq 1}$ converges to zero exponentially fast with *n* with an exponent dictated by D(P∥Q)≥0. Interestingly, the error exponent limit in (7) is insensitive to the magnitude of the fixed constraint ϵ>0 (the Type I error restriction).

The asymptotic result in (7) might change if we impose a setting with a monotonically decreasing sequence of Type I error restrictions. This HT scenario is relevant when a designer wants to analyze conditions where both errors tend to zero as the amount of evidence (parameterized by *n*) increases. In this scenario, Han et al. [49] studied the case when the Type I error sequence has an exponentially decreasing behavior. Complementing this analysis, Nagakawa et al. [50] extended the study considering a more general family of a decreasing sequence of Type I error restrictions. This result is the following:

**Theorem** **1** ([50], Theorem 1)**.** *If ${\left(\epsilon_{n}\right)}_{n}$ is $𝒪\left(e^{-rn}\right)$ for some r∈(0,D(P∥Q)), then the solutions of* (4) *satisfy that:*

(8) $$\lim_{n\to \infty }-\frac{1}{n}\log\left(\beta_{n}\left(\epsilon_{n}\right)\right)=D\left(P_{t^{*}}∥Q\right)<D\left(P∥Q\right)$$

The fact that D(P∥Q) is strictly bigger than $D(P_{t^{*}}∥Q)$, stated in (8)*, was demonstrated by Blahut in* [51]*, where*

$$P_{t}\left(\left\{x\right\}\right)\equiv C_{t}{P(\left(\left\{x\right\}\right)}^{1-t}Q{\left(\left\{x\right\}\right)}^{t},$$

$C_{t}$ is a normalization constant and $t^{*}$ is the unique solution of $D(P_{t^{*}}∥P)=r$.

The existence and uniqueness of the solution of $D(P_{t^{*}}∥P)=r$ are discussed in [50].

An important implication of Theorem 1 is the following result:

**Corollary** **1** ([50], Corollary)**.** *Let us assume that ${\left(1/\epsilon_{n}\right)}_{n}$ is $o(e^{rn})$ for some r>0 and then the solutions of* (4) *satisfy that:*

$$\lim_{n\to \infty }-\frac{1}{n}\log\left(\beta_{n}\left(\epsilon_{n}\right)\right)=D\left(P∥Q\right).$$

On these findings, we make two observations:

- Corollary 1 states that the same error exponent obtained in the fixed Type II error setting of Stein’s lemma is obtained for a stringent family of problems where the Type I restriction is vanishing with *n*. This family is the rich collection of binary HT problems where ${\left(\epsilon_{n}\right)}_{n\geq 1}$ tends to zero at a sub-exponential rate.
- On the other hand, when the Type I error restriction tends to zero exponentially fast (in Theorem 1), the error exponent is strictly smaller than D(P∥Q), meaning that, performance-wise, this problem is significantly more complex than the setting where ${\left(\epsilon_{n}\right)}_{n}$ is constant with *n* (presented in Lemma 1).

The presented information-theoretic analysis of centralized binary HT is theoretically powerful but has an intrinsic practical limitation: it relies on a perspective of the problem valid when the sample size approaches infinity. In contrast, in a practical setting, the test designer can only access a finite number of observations. This observation motivates a non-asymptotic analysis of centralized HT that looks at performance queues for finite data length regimes. The following section will explore this non-asymptotic performance perspective using the presented error exponent results.

## 3. Finite-Length Analysis of Centralized HT

A non-asymptotic analysis provides a more realistic understanding of achievable HT performance in scenarios where the length of the evidence is finite. In the setting presented in this section, we focus on the presentation of non-asymptotic bounds for ${\left(\beta_{n}\left(\epsilon \right)\right)}_{n\geq 1}$. Let us begin with the following theorem by Strassen [52] for the case when $\epsilon_{n}=\epsilon >0$:

**Theorem** **2** ([52], Theorem 1)**.** *If ϵ∈(0,1), then for a sufficiently large n>0, it follows that*

(9) $$-\frac{\log(\beta_{n}\left(\epsilon \right))}{n}=D\left(P∥Q\right)+\sqrt{\frac{V(P∥Q)}{n}}\Phi^{-1}\left(\epsilon \right)+\frac{\log n}{2n}+𝒪\left(\frac{1}{n}\right),$$

*where*

$$V\left(P∥Q\right)\equiv \sum_{x\in X}P\left(\left\{x\right\}\right){\left[\log\left(\frac{P(\{x\})}{Q(\{x\})}\right)-D\left(P∥Q\right)\right]}^{2}.$$

*and Φ(·) is the standard cumulative distribution function.*

Theorem 2 shows that $\left|D\left(P∥Q\right)-\left(-\frac{1}{n}\log\left(\beta_{n}\left(\epsilon \right)\right)\right)\right|$ is $𝒪\left(\frac{1}{\sqrt{n}}\right)$ where D(P∥Q) represents the error exponent limit (from Lemma 1) and $\left(-\frac{1}{n}\log\left(\beta_{n}\left(\epsilon \right)\right)\right)$ the finite-length error expression. This means that the velocity of convergence of $-\frac{1}{n}\log\left(\beta_{n}\left(\epsilon \right)\right)$ (the optimal error for a finite *n*) to its known limit D(P∥Q) is polynomial as $\frac{1}{\sqrt{n}}$. Importantly, this non-asymptotic result offers a method to approximate $\beta_{n}\left(\epsilon \right)$ with its error exponent limit D(P∥Q) and V(P∥Q) (for a sufficiently large *n*).

Complementing this result, Espinosa et al. [40] recently provided concrete upper and lower bounds for the discrepancy between $-\frac{1}{n}\log\left(\beta_{n}\left(\epsilon_{n}\right)\right)$ and its information limit D(P∥Q) (from Corollary 1) when the sequence ${\left(\epsilon_{n}\right)}_{n\geq 1}$ tends to zero at a sub-exponential rate.

**Theorem** **3** ([40], Theorem 1)**.** *Let ${\left(1/\epsilon_{n}\right)}_{n}$ be $o(e^{rn})$ for some r>0. Then, eventually in n, it follows that the solutions of* (4) *satisfy that:*

(10) $$\begin{array}{c} -\frac{1}{n}\log\left(\beta_{n}\left(\epsilon_{n}\right)\right)\geq D\left(P∥Q\right)-C_{X}\left(P,Q\right)\sqrt{\frac{2\ln(1/\epsilon_{n})}{n}} \end{array}$$

(11) $$\begin{array}{c} -\frac{1}{n}\log\left(\beta_{n}\left(\epsilon_{n}\right)\right)\leq D\left(P∥Q\right)+\frac{\log\left(\frac{1}{1-\epsilon_{n}-\delta_{n}}\right)}{n}+\delta_{n} \end{array}$$

*where*

$$C_{X}\left(P,Q\right)\equiv \underset{x\in X}{\sup}\left|\log\left(\frac{P(\{x\})}{Q(\{x\})}\right)\right|$$

*and $\delta_{n}\equiv C_{X}\left(P,Q\right)\sqrt{\frac{2\ln(1/\epsilon_{n})}{n}}$.*

A few observations about Theorems 2 and 3:

1. Theorem 3 establishes a non-asymptotic convergence rate for the Type II error when we impose a vanishing condition on ${\left(\epsilon_{n}\right)}_{n}$ that is sub-exponential. The bounds for the discrepancy $-\frac{1}{n}\log\left(\beta_{n}\left(\epsilon_{n}\right)\right)$ in this case are a function of the sequence ${\left(\epsilon_{n}\right)}_{n}$.
2. It is worth noting that the dependency on ${\left(\epsilon_{n}\right)}_{n}$ observed in these non-asymptotic results (in particular Theorem 3) is non-observed in the asymptotic limit in Corollary 1, which is D(P∥Q) as long as ${\left(1/\epsilon_{n}\right)}_{n}$ is sub-exponential.
3. The proof of Theorem 3 follows a standard information-theoretic approach with two parts: a constructive and an infeasibility argument (the details are presented in [40]).
4. If a fixed value of $\epsilon_{n}=\epsilon \in \left(0,1\right)$ is considered, Theorem 3 recovers the rate of convergence for the Type II error presented in Theorem 2.

To summarize, in light of the results covered in this section, along with some numerical evidence presented in [40], the idea that the expression $e^{-nD(P||Q)}$ serves as an adequate proxy for the probability of error when *n* is finite is highlighted. This is a very important point because it allows for the approximation of the Type II error (that might be difficult to compute from solving (4)) using an analytical error exponent expression. This observation is crucial because it enables the estimation of finite-length Type II errors using asymptotic error exponent results.

### 3.1. Other Finite-Length Results

Some other important results are worth mentioning. Polyanskiy–Poor–Verdú [53] (Theorem 52) established a stronger version of Theorem 2 by giving an explicit form to the $𝒪\left(\frac{1}{n}\right)$ term in (9). Another interesting refinement of Theorem 2 is proposed by Tan in Ref. [6]. This extension involves the universal bound of Berry–Esseen [54], which is a refinement of the central limit theorem because it specifies the rate at which this convergence takes place. Then, this result makes it possible to express the argument of $\Phi^{-1}\left(\epsilon \right)$ in a more refined way using the first- and second-order statistics of the likelihood ratio. Finally, non-asymptotic bounds for the exponential case (i.e., ${\left(1\epsilon_{n}\right)}_{n}$ is $𝒪\left(e^{rn}\right)$ for some r>0) was presented by Hoeffding in Ref. [55]. Although his result is not tight (order 𝒪(n)), his expansion is still relevant to produce finite-length interpretations.

### 3.2. Test against Independence

An emblematic BHT problem is when $P=P_{XY}$ is a joint (non-product) distribution between two rvs. *X* and *Y* and $Q=P_{X}\cdot P_{Y}$, i.e., $Q_{X,Y}$ is the product of the marginals of $P_{X,Y}$. Here, the BHT problem in (1) reduces to a test against independence [1] where we have that:

(12) $$\begin{array}{cc} D(P_{XY}∥Q_{XY})= & \sum_{(x,y)\in X\times Y}P_{XY}\left(\left\{\left(x,y\right)\right\}\right)\log\frac{P_{XY}\left(\left\{\left(x,y\right)\right\}\right)}{P_{X}\left(\left\{x\right\}\right)\cdot P_{Y}\left(\left\{y\right\}\right)}\\ \; & =I(X;Y)>0. \end{array}$$

I(X;Y) in (12) is the mutual information between *X* and *Y* [1]. Deciding about $H_{0}$ and $H_{1}$ from $\left(X_{1}^{n},Y_{1}^{n}\right)$ is a particular case of the centralized BHT studied in this section. Consequently, its error exponent (for the Type II error given a fixed Type I error restriction) is I(X;Y), which is a direct application of *Stein’s lemma* (Lemma 1). More details of this important BHT problem will be covered in Section 6.

### 3.3. Composite Hypothesis Testing

Hypothesis testing (HT) can be broadly classified into simple and composite HT. In simple HT, both the null and alternative hypotheses are completely specified, meaning there are no unknown parameters. In contrast, composite HT involves hypotheses that include unknown parameters, typically a collection of possible models for each hypothesis. In this composite setting, a key work for determining error exponents for the finite-alphabet case was developed by Hoeffding [55] using the generalized likelihood ratio test (GLRT) [12], which provides an optimal error exponent of the form:

(13) $$\underset{P\in \Pi }{\inf}D\left(P||Q\right),$$

where Π⊆P(X) is a class of probability distributions. Extensions of error exponents for small sample sizes (i.e., where the number of samples *n* is smaller than the size of the alphabet of the problem) and extensions to arbitrary distributions can be found in [56,57].

## 4. Distributed Hypothesis Testing

The general focus of this section is motivated by the problem of distributed detection under communication constraints [14,49,58]. In this context, researchers have actively studied the derivation of performance limits and the characterization of statistical properties of optimal detectors. Here, we highlight some significant results that can be seen as a progression of the centralized HT results presented in Section 2 and Section 3.

We will present the case of two information sources or observations, *X* and *Y*, located remotely one from the other. Formally, let us consider a finite-alphabet product space Z=X×Y, where P(Z) denotes the family of probabilities on Z. We have a random vector Z=(X,Y) with values in Z and equipped with a joint probability $P_{XY}\in P\left(Z\right)$. $P_{X}\in P\left(X\right)$ and $P_{Y}\in P\left(Y\right)$ denote the marginal of *X* and *Y*, respectively. $X_{1}^{n}=\left(X_{1},\ldots ,X_{n}\right)$ and $Y_{1}^{n}=\left(Y_{1},\ldots ,Y_{n}\right)$ represent the finite-block vectors with product (i.i.d.) distribution $P_{X,Y}^{n}\equiv P_{X_{1}^{n}Y_{1}^{n}}\in P\left(X^{n}\times Y^{n}\right)$. We consider two cases for $\left(X_{1}^{n},Y_{1}^{n}\right)$:

(14) $$\begin{array}{cc} H_{0}: & \left(X_{1}^{n},Y_{1}^{n}\right)∼P_{XY}^{n},\\ H_{1}: & \left(X_{1}^{n},Y_{1}^{n}\right)∼Q_{XY}^{n}, \end{array}$$

In (14), we assume the non-trivial condition that $D(P_{XY}∥Q_{XY})>0$.

At this point, we add a distinctive dimension to the HT task assuming that the decision agent does not have access to $\left(X_{1}^{n},Y_{1}^{n}\right)$; instead, it can be informed indirectly about $\left(X_{1}^{n},Y_{1}^{n}\right)$ (using an encoder) with a prescribed communication (in bits per sample) constraint. This scenario will be referred as distributed HT. Then, we will focus on the challenging problem of distributed binary HT under communication constraints in bits per sample. Adding this rate constraint dimension in the setting changes the analysis of the HT task significantly, as the test and its performance will be affected by this communication restriction.

### 4.1. The Rate-Constrained Setup

**Figure 1:**
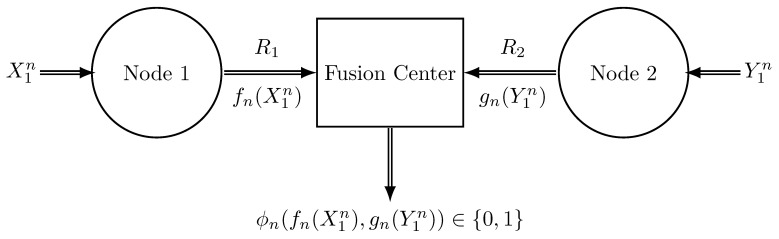
The general distributed test. $f_{n}\left(\cdot \right)$ and $g_{n}\left(\cdot \right)$ are the two encoders and $\phi_{n}\left(\cdot \right)$ represents the detector (decision-maker).

Let us introduce the specific decentralized HT problem. We have three agents: Node 1, Node 2, and the fusion center. Node 1 and Node 2 are equipped with an encoder each (denoted by $f_{n}\left(\cdot \right)$ and $g_{n}\left(\cdot \right)$, respectively). They need to communicate to the fusion center a finite-rate description of $X_{1}^{n}$ and $Y_{1}^{n}$, respectively. The fusion center must decide the true underlying hypothesis ($H_{0}$ or $H_{1}$) from a lossy (encoded) version of the joint vector that we denote by $\left(f_{n}\left(X_{1}^{n}\right),g_{n}\left(Y_{1}^{n}\right)\right)$. The process is illustrated in Figure 1.

The decision rule is represented by two encoders $\left(f_{n},g_{n}\right)$ of rates $R_{1}$ and $R_{2}$ (in bits per sample), respectively, and a decoder $\phi_{n}$ of length *n* where:

(15) $$\begin{array}{cc} \; & f_{n}:X^{n}\to \left\{1,\ldots ,2^{nR_{1}}\right\},\;\left(\mathrm{encoder}1\right)\\ \; & g_{n}:Y^{n}\to \left\{1,\ldots ,2^{nR_{2}}\right\},\;\left(\mathrm{encoder}2\right)\\ \; & \phi_{n}:\left\{1,\ldots ,2^{nR_{1}}\right\}\times \left\{1,\ldots ,2^{nR_{2}}\right\}\to \left\{0,1\right\},\;\left(\mathrm{decoder}\right). \end{array}$$

$f_{n}\left(\cdot \right)$ and $g_{n}\left(\cdot \right)$ produce a fixed-rate lossy version (or quantization) of $X_{1}^{n}$ and $Y_{1}^{n}$, respectively, and $\phi_{n}\left(\cdot \right)$ represents the detector (or classifier) acting on the two-sided compressed data $\left(f_{n}\left(X_{1}^{n}\right),g_{n}\left(Y_{1}^{n}\right)\right)\in \left\{1,\ldots ,2^{nR_{1}}\right\}\times \left\{1,\ldots ,2^{nR_{2}}\right\}$ as illustrated in Figure 1.

The corresponding Type I and Type II error probabilities are [42,59]:

(16) $$\begin{array}{cc} P_{0}\left(f_{n},g_{n},\phi_{n}\right) & \equiv P_{XY}^{n}\left(A^{c}\left(f_{n},g_{n},\phi_{n}\right)\right)\;\mathrm{and}\; \end{array}$$

(17) $$\begin{array}{cc} P_{1}\left(f_{n},g_{n},\phi_{n}\right) & \equiv Q_{XY}^{n}\left(A(f_{n},g_{n},\phi_{n})\right), \end{array}$$

where $A\left(f_{n},g_{n},\phi_{n}\right)\equiv \left\{\left(x_{1}^{n},y_{1}^{n}\right)\in X^{n}\times Y^{n}:\phi_{n}\left(f_{n}\left(x_{1}^{n}\right),g_{n}\left(y_{1}^{n}\right)\right)=0\right\}$. For any ϵ>0, we are interested in solving:

(18) $$\beta_{n}\left(\epsilon ,R_{1},R_{2}\right)\equiv \min_{\left(f_{n},g_{n},\phi_{n}\right)}\left\{P_{1}\left(f_{n},g_{n},\phi_{n}\right):P_{0}\left(f_{n},g_{n},\phi_{n}\right)\leq \epsilon \right\},$$

where the minimum in (18) is over all the encoders–decoders of the form presented in (15). It is worth noting that the expression $\beta_{n}\left(\epsilon ,R_{1},R_{2}\right)$, which represents the optimization in (18), is a function of the underlying models, $P_{XY}$ and $Q_{XY}$, as well as the operational constraints parametrized by $\left(R_{1},R_{2},\epsilon \right)$.

As in the results presented in Section 2 for the centralized HT problem, it is important to derive asymptotic (and single-letter—a single-letter characterization refers to an expression that is a function of a single variable representation of the models, which in this case is a functional expression of $P_{X,Y}$ and $Q_{X,Y}$) expressions for the convergence of $\beta_{n}\left(\epsilon ,R_{1},R_{2}\right)$ in (18) as *n* tends to infinity.

### 4.2. Asymptotic Results

The derivation of an error exponent expression for ${\left(\beta_{n}\left(\epsilon ,R_{1},R_{2}\right)\right)}_{n}$ is traditionally divided into two technical parts: the creation of an achievable encoder–decoder construction that meets the operational constraints of the problem (or achievable argument) and an unfeasible (impossibility) result (or converse argument). On the first technical part (achievable argument), one of the most general and recognized results for the distributed HT was established by Han in Ref. [60]. This result presents a single-letter lower bound for the error exponent of the general HT setting described in Figure 1:

**Theorem** **4** ([60], Theorem 7)**.** *For the distributed HT problem, let us consider two joint distributions $P_{XY}$ and $Q_{XY}$ defined in* (14) *and $R_{1},R_{2}>0$. Under $H_{0}$, consider two additional random variables U,V such that U ―○ X ―○ Y ―○ V forms a Markov chain and denote its joint distribution $P_{UXYV}=P_{U|X}P_{V|Y}P_{XY}$ and marginals $P_{UV}$ and $P_{XY}$. For $P_{UV}\in P\left(U\times V\right)$, denote $L\left(P_{UV}\right)\subset P\left(U\times V\times X\times Y\right)$ and $S\left(R_{1},R_{2}\right)\subset P\left(U\times V\right)$ as the sets of probabilities defined by*

(19) $$\begin{array}{cc} L(P_{UV}) & \equiv \left\{\mu_{UXYV}\in P\left(U\times X\times Y\times V\right)\;\mu_{UX}=P_{UX}\;,\;\mu_{VY}\equiv P_{VY},\;\mu_{UV}=P_{UV}\right\} \end{array}$$

(20) $$\begin{array}{cc} S(R_{1},R_{2}) & =\left\{P_{UV}\in P\left(U\times V\right):U\;―\;○\;X\;―\;○\;Y\;―\;○\;V,\;s.t.\;I\left(U;X\right)\leq R_{1},I\left(V;Y\right)\leq R_{2}\;\right\}. \end{array}$$

*Then, it follows that*

(21) $$\begin{array}{cc} \; & \lim_{n\to \infty }-\frac{1}{n}\log\beta_{n}\left(\epsilon ,R_{1},R_{2}\right)\geq \max_{P_{UV}\in S\left(R_{1},R_{2}\right)}\;\min_{\mu_{UXYV}\in L\left(P_{UV}\right)}D\left(\mu_{UXYV}\left|\right|Q_{UXYV}\right)\;, \end{array}$$

*where $Q_{UXYV}$ is such that $Q_{UXYV}\equiv P_{U|X}P_{V|Y}Q_{XY}$.*

This is an achievable result that is significant as it establishes a lower bound for the exponential rate of convergence of ${\left(\beta_{n}\left(\epsilon ,R_{1},R_{2}\right)\right)}_{n}$ in (21). Importantly, the result indicates that the sequence ${\left(\beta_{n}\left(\epsilon ,R_{1},R_{2}\right)\right)}_{n}$ tends to zero exponentially fast in the same way as observed in the classical centralized HT problem (see Section 2.2). The analytical lower bound expression in (21) is also a single-letter characterization of the problem as the results presented in Section 2.2.

On the specific expression of the error exponent for this distributed HT task, to this date, there is no formal result that proves that the expression in the LHS of (21) is optimal (i.e., tight) and it remains an open conjecture to prove or disprove the optimality of the bound in (21).

It is worth mentioning that the existing results in the distributed HT context involve high technicalities due to the complex nature of the distributions induced by the encoders ($f_{n}$ and $g_{n}$) of this rate-restricted setting. Indeed, as the results presented in Theorem 4, many results are provided in the form of a lower bound for the error exponent (i.e., an achievable construction) without guaranteeing that the proposed encoder–decoder schemes used in that achievable argument are optimal.

**Figure 2:**
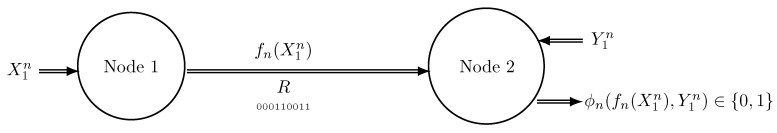
The one-directional distributed test. $f_{n}\left(\cdot \right)$ represents the encoder and $\phi_{n}\left(\cdot \right)$ is the detector.

An interesting and well-considered case of the setting presented in this section corresponds to the scenario when one of the nodes is fully observed (for example, when $R_{2}>H\left(Y\right)$). This distributed HT case is referred to as unidirectional, which is illustrated in Figure 2. This one-sided system allows for direct access to the vector $Y_{1}^{n}$. In the following section, we will analyze this special case.

## 5. The One-Sided Distributed HT

In the one-sided distributed context illustrated in Figure 2, the decision rule consists of a pair of an encoder and a decoder $\left(f_{n},\phi_{n}\right)$ of length *n* and rate *R*, where $f_{n}\left(\cdot \right)$ has the following restriction in bits per sample:

(22) $$\begin{array}{cc} \; & f_{n}:X^{n}\to \left\{1,\ldots ,2^{nR}\right\},\;\left(\mathrm{encoder}\right)\\ \; & \phi_{n}:\left\{1,\ldots ,2^{nR}\right\}\times Y^{n}\to \Theta =\left\{0,1\right\},\;\left(\mathrm{decoder}\right). \end{array}$$

$f_{n}\left(\cdot \right)$ induces a lossy description (or quantization) of $X_{1}^{n}$, and $\phi_{n}\left(\cdot \right)$ is the detector (or classifier) acting on the one-sided compressed data $\left(f_{n}\left(X_{1}^{n}\right),Y_{1}^{n}\right)\in \left\{1,\ldots ,2^{nR}\right\}\times Y^{n}$.

The encoder functions as a remote agent that captures $X_{1}^{n}$ and transmits a finite description (using *R* bits per sample) of $X_{1}^{n}$ to a fusion center (see Figure 2). The fusion center receives the quantization of $X_{1}^{n}$ and simultaneously senses a second modality $Y_{1}^{n}$ to estimate the true distribution of the joint vector $\left(X_{1}^{n},Y_{1}^{n}\right)$ using $\phi_{n}\left(\cdot \right)$. For any pair $\left(f_{n},\phi_{n}\right)$, we have their corresponding Type I and Type II error probabilities [42,59]:

(23) $$\begin{array}{cc} P_{0}\left(f_{n},\phi_{n}\right) & \equiv P_{XY}^{n}\left(A^{c}\left(f_{n},\phi_{n}\right)\right)\;\mathrm{and}\; \end{array}$$

(24) $$\begin{array}{cc} P_{1}\left(f_{n},\phi_{n}\right) & \equiv Q_{XY}^{n}\left(A(f_{n},\phi_{n})\right). \end{array}$$

Here, we have that $A\left(f_{n},\phi_{n}\right)\equiv \left\{\left(x_{1}^{n},y_{1}^{n}\right)\in X^{n}\times Y^{n}:\phi_{n}\left(f_{n}\left(x_{1}^{n}\right),y_{1}^{n}\right)=0\right\}$.

As before, for any ϵ>0, we are interested in solving the following operational problem:

(25) $$\beta_{n}\left(\epsilon ,R\right)\equiv \min_{\left(f_{n},\phi_{n}\right)}\left\{P_{1}\left(f_{n},\phi_{n}\right):P_{0}\left(f_{n},\phi_{n}\right)\leq \epsilon \right\}.$$

The following result can be interpreted as the specialized version of Theorem 4, in the one-sided distributed HT setting of Figure 2, offering a positive lower bound (by an achievable construction) for the error exponent limit $\lim_{n\to \infty }-\frac{1}{n}\log\beta_{n}\left(\epsilon ,R\right)$:

**Theorem** **5** ([60], Theorem 2)**.** *Consider R>0, and under $H_{0}$, let us consider an additional random variable U such that U ―○ X ―○ Y forms a Markov chain and denote its joint distribution by $P_{UXY}=P_{U|X}P_{XY}$ with marginals $P_{UX}$ and $P_{UY}$. For $P_{UV}\in P\left(U\times V\right)$, let us denote $L\left(P_{U}\right)\subset P\left(U\times X\times Y\right)$ and S(R)⊂P(U) as the sets of probability defined by*

(26) $$\begin{array}{cc} S(R) & \equiv \left\{P_{U}\in P\left(U\right):U\;―\;○\;X\;―\;○\;Y,\;\;s.t.\;\;I\left(U;X\right)\leq R\right\}\;, \end{array}$$

(27) $$\begin{array}{cc} L(P_{U}) & \equiv \left\{\mu_{UXY}\in P\left(U\times X\times Y\right)\;:\mu_{UX}=P_{UX}\;,\;\mu_{UY}=P_{UY}\right\}\;. \end{array}$$

*Then, it follows that the solutions of* (25) *satisfy that:*

(28) $$\begin{array}{cc} \; & \lim_{n\to \infty }-\frac{1}{n}\log\beta_{n}\left(\epsilon ,R\right)\geq \max_{P_{U}\in S\left(R\right)}\;\min_{\mu_{UXY}\in L\left(P_{U}\right)}D\left(\mu_{UXY}\left|\right|Q_{UXY}\right)\;, \end{array}$$

*where $Q_{UXY}$ is such that $Q_{UXY}\equiv P_{U|X}Q_{XY}$ forms a Markov chain with $Q_{XY}$.*

Again, this result provides a valuable information-theoretic lower bound for the error exponent of this distributed one-sided HT setting. Comparing Theorem 5 with Theorem 4, we see that both bounds correspond to finding the best representations over the possible encoders of *X* and *Y* (given by *U* and *V*, respectively). In this way, it is evident that Theorem 5 is a simplified version of Theorem 4 because there is no restriction (and therefore no encoder) for the variable *Y* in the one-sided setting.

As in the result presented in Theorem 4, it remains an open problem to prove the conjecture that the single-letter expression in Equation (28) is optimal. Interestingly, an optimal error exponent expression for the case of a one-sided test against independence (i.e., when $Q_{XY}=P_{X}\cdot P_{Y}$) exists, which is the main focus of the next section.

## 6. Distributed Test against Independence

As pointed out in Section 5, an interesting problem is a case where the alternative hypothesis corresponds to the product of the marginals (i.e., a test against independence, $Q_{XY}=P_{X}\cdot P_{Y}$). The test against independence holds significant importance in theory and for practical applications [61,62,63,64,65,66,67]. It is a fundamental tool for detecting and quantifying relationships between variables, and predictive modeling tasks, where identifying dependencies or redundancies is essential for accurate and reliable predictions [23,24,27,65,66]. In this section, we will analyze this particular test in the context of the one-sided HT setting in Figure 2. We will cover the fundamental performance limit that characterizes in a closed form its error exponent limit and some recent non-asymptotic results, and we will analyze its surprising connections with the information bottleneck (IB) problem [68].

### 6.1. Asymptotic Results

Ahlswede and Csiszár in [14] determined the following fundamental result:

**Theorem** **6** ([14], Theorem 3)**.** *For any ϵ>0, it follows that the solutions of* (25) *when $Q_{X,Y}=P_{X}\cdot P_{Y}$ satisfy that:*

(29) $$\xi \left(R\right)\equiv \lim_{n\to \infty }-\frac{1}{n}\log\beta_{n}\left(\epsilon ,R\right)=\max_{\begin{array}{c} P_{U}:U\;―\;○\;X\;―\;○\;Y\\ I(U;X)\leq R\;|U|\leq |X|+1 \end{array}}I\left(U;Y\right),$$

*where U ―○ X ―○ Y.*

Important remarks about Theorem 6

- In contrast to the two previous error exponent lower bounds in Theorems 4 and 5, this result does present a single-letter closed-form expression (function of $P_{X,Y}$) of the precise exponential rate of convergence of the error sequence ${\left(\beta_{n}\left(\epsilon ,R\right)\right)}_{n}$ for a fixed (with *n*) Type I error constraint. The dependency (sensibility) on R>0 is clear in (29) as well as its invariance to ϵ>0.
- On the role of *R* (the communication constraint), the optimization presented in (29) presents a formal trade-off between representation quality I(U;Y) and compression I(U;X) in the sense of finding the best lossy encoder of *X* for predicting *Y* (in the MI sense) given a compression constraint of the form I(U;X)≤R.
- This result reveals an interesting link to the problem of noisy lossy source coding using the log-loss fidelity function [69]. Specifically, the performance limits on the right-hand side (RHS) of (29) exactly match the distortion-rate function of the information bottleneck (IB) problem [68]. This link will be explored in more detail in Section 8.1.
- Finally, this significant asymptotic result can be viewed as an extension of *Stein’s lemma* in the decentralized scenario illustrated in Figure 2.

A step forward in this error exponent analysis is to look at more stringent distributed HT settings where ${\left(\epsilon_{n}\right)}_{n}$ tends to zero with *n*. In this direction, the author presented a first result in [49], which is a lower bound for the error exponent of the Type II error in the case of exponentially decreasing Type I error restrictions:

**Proposition** **1** ([49], Corollary 2). *If $\epsilon_{n}\leq e^{-rn}$ for some r>0, then the solutions of* (25) *when $Q_{X,Y}=P_{X}\cdot P_{Y}$ satisfy that:*

(30) $$\begin{array}{cc} \underset{n\to \infty }{lim inf}-\frac{1}{n}\log\beta_{n}\left(\epsilon_{n},R\right)\geq & \max_{\mu_{U|X}\in \rho \left(R,r\right)}\min_{\begin{array}{c} {\tilde{P}}_{UXY}\in P\left(U\times X\times Y\right)\\ D({\tilde{P}}_{UXY}∥P_{UXY})\leq r\\ {\tilde{P}}_{U|X}=P_{U|X}=\mu_{U|X}\\ U\;―\;○\;X\;―\;○\;Y \end{array}}\left[D\left({\tilde{P}}_{X}∥P_{X}\right)+I\left(U;Y\right)\right], \end{array}$$

*where*

$$\rho \left(R,r\right)\equiv \{\mu_{U|X}\in P\left(U|X\right)|\max_{\begin{array}{c} {\tilde{P}}_{X}:D\left({\tilde{P}}_{X}∥Q_{X}\right)\leq r\\ {\tilde{P}}_{U|X}=\mu_{U|X}\\ P_{UX}=\mu_{U|X}\cdot {\tilde{P}}_{X} \end{array}}I\left(U;X\right)\leq R\}.$$

*In this last notation, P(U|X) is the collection of conditional probabilities from X to U.*

Complementing Proposition 1, in the sub-exponential vanishing regime for the restriction sequence ${\left(\epsilon_{n}\right)}_{n}$ (of the Type I error probability), the counterpart of what is known in the centralized HT setting (in Section 2) when comparing the error exponents obtained in Lemma 1 and Corollary 1 is obtained with the following result:

**Theorem** **7** ([15], Theorem 1)**.** *If ${\left(1/\epsilon_{n}\right)}_{n}=o\left(e^{rn}\right)$ for some r>0, then we have that:*

(31) $$\lim_{n\to \infty }-\frac{1}{n}\log\left(\beta_{n}\left(\epsilon_{n},R\right)\right)=\xi \left(R\right).$$

*The expression ξ(R) in* (31) *is presented in* (29).

This last result determines an extensive regime on the velocity at which ${\left(\epsilon_{n}\right)}_{n}$ tends to zero for which the error exponent of the one-side test against independence matches the expression obtained for the less restrictive setting (when $\epsilon_{n}=\epsilon >0$) in Theorem 6. The significance of this result lies in the fact that before it was established, there was no assurance that the asymptotic limit in (31) would match the result in Theorem 6: remember that Theorem 7 is looking at vanishing regimes for ${\left(\epsilon_{n}\right)}_{n}$.

### 6.2. Finite-Length Results

An important class of new results for the task of a distributed test against independence is non-asymptotic [15]. This set of results bounds the error expression $\beta_{n}\left(\epsilon_{n},R\right)$ in (25). Crucially, these results show that it is possible to produce relevant finite-length performance bounds for $\beta_{n}\left(\epsilon_{n},R\right)$ from the asymptotic error exponent limits presented in Section 6.1. In this exposition, we cover the more challenging and rich case when ${\left(\epsilon_{n}\right)}_{n}$ tends to zero (as the sample size grows) with different velocities that can then be used to recover the simpler (standard) case when $\epsilon_{n}=\epsilon$. In this analysis, we will show how the operational restriction on the restriction sequence ${\left(\epsilon_{n}\right)}_{n}$ affects the performance bounds derived for $\beta_{n}\left(\epsilon_{n},R\right)$ in conjunction with other problem elements.

Specifically, the following result (Theorem 8) studies the gap between $\beta_{n}\left(\epsilon_{n},R\right)$ and its exponential approximation $e^{-n\xi (R)}$ (extrapolating the result in Theorems 6 and 7 to a finite-sample regime) under different scenarios for the vanishing Type I error restriction. As a corollary, the results also determine the velocity at which $-\frac{1}{n}\log\beta_{n}\left(\epsilon_{n},R\right)$ converges to its limit in (31), richly improving the asymptotic findings presented in Section 6.1.

**Theorem** **8** ([15], Theorem 2)**.** *Let us assume that P≪Q and R<H(X). Let us define*

$$C\left(P_{XY}\right)\equiv \underset{(x,y)\in X\times Y}{\sup}|\log\left(\frac{P_{XY}\left(\left\{\left(x,y\right)\right\}\right)}{Q_{XY}\left(\left\{\left(x,y\right)\right\}\right)}\right)|<\infty .$$

*We have the following set of results for $\beta_{n}\left(\epsilon_{n},R\right))$ when $Q_{X,Y}=P_{X}\cdot P_{Y}$:*

*(i)*  *If ${\left(\epsilon_{n}\right)}_{n}={\left(1/\log\left(n\right)\right)}_{n}$ (logarithmic), then*
(32) $$-\frac{1}{n}\log\left(\beta_{n}\left(\epsilon_{n},R\right)\right)-\xi \left(R\right)\geq \left(\frac{dD(R)}{6dR}-\frac{\sqrt{2\ln(\log(n))}C\left(P_{XY}\right)}{\log(n)}-o\left(1\right)\right)\frac{\log n}{n^{1/3}}$$
  *and*
(33) $$-\frac{1}{n}\log\left(\beta_{n}\left(\epsilon_{n},R\right)\right)-\xi \left(R\right)\leq \left(16C\left(P_{XY}\right)+\frac{\log\left(\log\left(n\right)\right)\sqrt{\log(n)}}{n}\right)\frac{1}{\sqrt{\log(n)}}.$$
*(ii)*  *If ${\left(\epsilon_{n}\right)}_{n}={\left(1/n^{p}\right)}_{n}$ (polynomial) with 2>p>0, then*
(34) $$-\frac{1}{n}\log\left(\beta_{n}\left(\epsilon_{n},R\right)\right)-\xi \left(R\right)\geq \left(\frac{1}{6}\frac{dD(R)}{dR}-\frac{\sqrt{2p\ln(n)}}{\log n}C\left(P_{XY}\right)-o\left(1\right)\right)\frac{\log n}{n^{1/3}}$$
  *and*
(35) $$-\frac{1}{n}\log\left(\beta_{n}\left(\epsilon_{n},R\right)\right)-\xi \left(R\right)\leq \left(16C\left(P_{XY}\right)+\frac{p\log(n)}{n^{1-p/2}}\right)\frac{1}{n^{p/2}}.$$
*(iii)*  *If ${\left(\epsilon_{n}\right)}_{n}={\left(1/n^{p}\right)}_{n}$ (polynomial) with p≥2, then*
(36) $$-\frac{1}{n}\log\left(\beta_{n}\left(\epsilon_{n},R\right)\right)-\xi \left(R\right)\geq \left(\frac{1}{6}\frac{dD(R)}{dR}-\frac{\sqrt{2p\ln(n)}}{\log n}C\left(P_{XY}\right)-o\left(1\right)\right)\frac{\log n}{n^{1/3}}$$
  *and*
(37) $$-\frac{1}{n}\log\left(\beta_{n}\left(\epsilon_{n},R\right)\right)-\xi \left(R\right)\leq \left(8\sqrt{2}C\left(P_{XY}\right)\frac{\sqrt{n^{2-p}+1}}{\log(n)}+2\right)\frac{\log(n)}{n}.$$
*(iv)*  *If ${\left(\epsilon_{n}\right)}_{n}={\left(1/e^{n^{p}}\right)}_{n}$ (superpolynomial) with p∈(0,1), then*
(38) $$-\frac{1}{n}\log\left(\beta_{n}\left(\epsilon_{n},R\right)\right)-\xi \left(R\right)\geq \left(\frac{\left(1-p\right)}{6}\frac{dD(R)}{dR}-\frac{\sqrt{2}C\left(P_{XY}\right)}{\log(n)}-o\left(1\right)\right)\frac{\log n}{n^{(1-p)/3}}$$
  *and*
(39) $$-\frac{1}{n}\log\left(\beta_{n}\left(\epsilon_{n},R\right)\right)-\xi \left(R\right)\leq \left(8\sqrt{2}C\left(P_{XY}\right)\frac{\sqrt{e^{-n^{p}}n^{2}+1}}{\log(n)}+2\right)\frac{\log(n)}{n}.$$

*In all these results, D(R) is the well-known rate-distortion function* [1]*—for completeness, Appendix A offers a brief presentation of the rate-distortion function.*

A few technical comments about the derivation and interpretations of these bounds:

- Regarding the upper bounds of $-\frac{1}{n}\log\left(\beta_{n}\left(\epsilon_{n},R\right)\right)$ presented in (33), (35), (37), and (39), these are obtained from an impossibility argument (or converse argument). In these bounds, we observe that as ${\left(\epsilon_{n}\right)}_{n}$ goes to zero faster (from case to case), the speed at which the bound tends to zero increases from the slower rate $𝒪\left(1/\sqrt{\log(n)}\right)$ in (33) to the faster rate $𝒪\left(\log(n)/n\right)$ in (39). Therefore, by imposing a more restrictive condition on ${\left(\epsilon_{n}\right)}_{n}$, there is a noticeable effect in the velocity of convergence of the bounds obtained from this upper-bound perspective.
- Regarding the lower bounds of $-\frac{1}{n}\log\beta_{n}\left(\epsilon_{n},R\right)$ presented in (32), (34), (36), and (38), these are obtained from an achievable argument (which is a concrete encoder–decoder construction). In these bounds, as ${\left(\epsilon_{n}\right)}_{n}$ goes faster to zero (from case to case), we see that the speed of this discrepancy is not affected for the logarithmic and polynomial cases, but the constants involved in those bounds change to smaller magnitudes. On the other hand, the derived expression for the superpolynomial case decreases in the speed at which the discrepancy, with the error exponent (i.e., $-\frac{1}{n}\log\left(\beta_{n}\left(\epsilon_{n},R\right)\right)-\xi \left(R\right)$), tends to zero. These trends align with the observation that relaxing the speed of ${\left(\epsilon_{n}\right)}_{n}$ makes the decision problem less restrictive, allowing for the possibility of achieving a better (smaller) Type II error than that predicted by the asymptotic limit, $e^{-n\xi (R)}$.

## 7. Collaboration in Distributed HT

Finally, we explore the role that collaboration plays in distributed HT to improve performance. On this, we cover some results for distributed testing against independence. Collaboration in this context refers to a decentralized detection scenario in which two agents cooperate by exchanging messages within a specific rate constraint (in bits per sample) to make a final decision. This setup is relevant in cooperative communication systems, where each wireless agent (user) transmits its own data and serves as a cooperative agent for other users. In this scenario, each agent transmits its own information (bits) and additional data for its partner to meet an operational requirement [70,71].

### 7.1. The Setup

**Figure 3:**
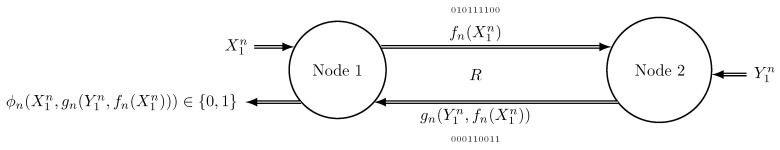
The one-round distributed test. $f_{n}\left(\cdot \right)$ and $g_{n}\left(\cdot \right)$ represent the encoders and $\phi_{n}\left(\cdot \right)$ is the detector.

Under this umbrella, we consider a setup where Node 1 and Node 2 exchange messages (using encoders) before making a final decision (the decoder). In particular, we look at the one-round communication setting shown in Figure 3. In this scenario, we identify the role of two encoders $\left(f_{n}\left(\cdot \right),g_{n}\left(\cdot \right)\right)$ and a detector $\phi_{n}$ given by:

(40) $$\begin{array}{cc} f_{n} & :X^{n}⟶U,\;\left(encoder\;1\right)\\ g_{n} & :Y^{n}\times U⟶V,\;\left(encoder\;2\right)\\ \phi_{n} & :X^{n}\times V⟶\left\{0,1\right\},\;\left(detector\right). \end{array}$$

The two encoders $\left(f_{n}\left(\cdot \right),g_{n}\left(\cdot \right)\right)$ meet the following fixed-rate communication constraints in bits per sample:

(41) $$\log\left(|U||V|\right)\leq nR.$$

It is worth noticing the difference with the one-sided distributed setting depicted in Figure 2, where Node 1 transmits to Node 2 in a non-collaborative manner. Indeed, the collaboration scheme (in Figure 3) recovers the setting in Figure 2 as a particular case—when R>H(Y), see [38] for more details. Consequently, this observation opens the question of evaluating the benefit of collaboration given the same rate constraint.

Returning to the collaboration strategy in (40), given the encoders–decoder $(f_{n}\left(\cdot \right)$, $g_{n}\left(\cdot \right)$, and $\phi_{n}\left(\cdot \right))$, data transmission occurs in two stages. In the first stage, $f_{n}\left(\cdot \right)$ sends information from Node 1 to Node 2. In the second stage, $g_{n}\left(\cdot \right)$ transmits information from Node 2 back to Node 1. Subsequently, Node 1 makes the final decision after receiving the message from Node 2, adhering to an overall rate constraint as specified in (41). The information flows bidirectionally, as depicted in Figure 3. It is important to note that the total bit budget for these two data compression stages is limited by an overall rate constraint R>0.

As for performance, the two (Type I and Type II) errors are:

(42) $$\begin{array}{cc} \alpha_{n}\left(f_{n},g_{n},\phi_{n}\right) & \equiv P_{XY}^{n}\left(A^{c}\left(f_{n},g_{n},\phi_{n}\right)\right)\;\mathrm{and}\; \end{array}$$

(43) $$\begin{array}{cc} \beta_{n}\left(f_{n},g_{n},\phi_{n}\right) & \equiv Q_{XY}^{n}\left(A(f_{n},g_{n},\phi_{n})\right). \end{array}$$

In these expressions, $A\left(f_{n},g_{n},\phi_{n}\right)\equiv \left\{\left(x_{1}^{n},y_{1}^{n}\right)\in X^{n}\times Y^{n}:\phi_{n}\left(x_{1}^{n},g_{n}\left(y_{1}^{n},f\left(x_{1}^{n}\right)\right)\right)=0\right\}$. In analogy to what was presented in (25), the optimal trade-off between Type I and Type II errors is given by

(44) $$\beta_{n}^{c}\left(\epsilon ,R\right)\equiv \min_{f_{n},g_{n},\phi_{n}}\left\{\beta_{n}\left(f_{n},g_{n},\phi_{n}\right):\alpha_{n}\left(f_{n},g_{n},\phi_{n}\right)\leq \epsilon \right\}.$$

As before, the minimization in (44) is over all the rules in (40) that satisfy the bit-rate constraint in (41).

### 7.2. An Asymptotic Result

Here, we present an analytical expression for the limit of $-\frac{1}{n}\log\beta_{n}^{c}\left(R,\epsilon \right)$ when *n* grows, similar to the results presented in Theorems 6 and 7. This result was obtained by Xiang [38] and is the following:

**Theorem** **9** ([38], Theorem 2)**.** *Given $P_{X,Y}$ and R>0, the optimal performances in* (44) *satisfy that:*

(45) $$\lim_{\epsilon \to 0}\;\lim_{n\to \infty }-\frac{1}{n}\log\beta_{n}^{c}\left(R,\epsilon \right)=E\left(R\right).$$

*In the result in* (45)*, E(R) is the solution of the following info-max optimization:*

(46) $$E\left(R\right)\equiv \max_{\begin{array}{c} P_{U|X}:X\mapsto P\left(U\right)\\ P_{V|UY}:U\times Y\mapsto P\left(V\right)\\ \;s.t.\;I(U;X)+I(V;Y|U)\leq R \end{array}}\left[I(U;Y)+I(V;X|U)\right].\;$$

Analysis of Theorem 9:

- In (46), $\left(X,Y\right)∼P_{XY}$ and *U* and *V* are two auxiliary random variables obtained from the joint vector $\left(X,Y,U,V\right)∼P_{X,Y}\cdot P_{U|X}\cdot P_{V|U,Y}$ meaning that
  (47) X ―○ (Y,U) ―○ V.
  In this notation, $P_{U|X}$ represents a conditional distribution from X to U, and $P_{V|U,Y}$ represents the conditional distributions from U×V to V.
- The expression in (45) indicates that $\beta^{c}n\left(\epsilon ,R\right)$ decreases exponentially with *n*, with an error exponent E(R)>0 fully determined by (46). Similar to the previous single-letter result, particularly the information bottleneck (IB) problem in (48), E(R) is derived from an information-driven optimization that depends on the model $P_{X,Y}$ (under $H_{0}$) and the operational constraint R>0. Unlike the result in Theorem 6, this asymptotic expression is obtained when considering an arbitrarily small Type I error restriction (parametrized by ϵ>0). Therefore, this result is more restrictive than previously presented error exponent results. Extending it to the non-vanishing case when ϵ>0 is still an open unsolved problem.
- When comparing the obtained error exponents, E(R) in (46) and its non-collaborative counterpart ξ(R) in (29), we notice that both are solutions to single-letter optimization dependent on $P_{X,Y}$ and R>0, and these optimizations are similar in the sense that their objective functions incorporate information measures with information (compression) constraints. Crucially, the single-letter task used to derive E(R) includes an additional non-zero term, I(V;X|U)>0, which is absent in the expression for ξ(R). This term, I(V;X|U)>0, arises from the capability of re-transmission, which is a unique aspect of the collaborative strategy. This additional (non-negative) information component provides a non-zero gain in the asymptotic error exponent, indicating that collaboration leads to an increase in performance, i.e., E(R)>ξ(R).

## 8. Applications

This section will explore practical aspects of the presented error exponents analysis and their concrete applications in different contexts. First, we will examine their connection with the information bottleneck method [68], demonstrating how error exponents can enhance data compression and feature extraction. Next, we will discuss their application in different network architectures, highlighting how error exponents can optimize encoding schemes and improve the reliability and efficiency of data transmission in communication systems. Finally, we will delve into a numerical analysis, showcasing the performance of error exponents in a distributed context and discussing how this can be implemented in a communication network.

### 8.1. Distributed HT and the Information Bottleneck Problem

It is worth noting that the asymptotic limit presented in Theorems 6 and 7 for the one-sided distributed test against independence, i.e., the solution of

(48) $$\xi \left(R\right)=\max_{\begin{array}{c} P_{U}:U\;―\;○\;X\;―\;○\;Y\\ I(U;X)\leq R\;|U|\leq |X|+1 \end{array}}I\left(U;Y\right),$$

corresponds exactly with the information bottleneck (IB) optimization problem [68]. The IB problem was introduced in [72] as a particular case of the celebrated rate-distortion function known in lossy compression [1,73]. Indeed, the optimization in (48) is exactly the rate-distortion function when the distortion is the log-loss function [74].

Recently, the IB problem in (48) has gained significant attention in machine learning. The IB has emerged as a relevant concept in machine learning, shedding light on the underlying mechanisms of learning and generalization. This optimization has been adopted in machine learning to learn expressive encoders (latent variables) from data [75,76,77,78]. This last representation learning task is closely connected with the encoder–decoder structure presented in this survey for the one-sided distributed HT task. At its core, the IB problem in (48) offers a principle to implement a trade-off between compression and prediction for an encoder–decoder design of learning systems. It asserts that for a learning algorithm to generalize effectively, it must balance preserving relevant information about the input data while discarding redundant or irrelevant details. By implementing the IB problem, it has been shown that learning algorithms can achieve better generalization by compressing the input data into a compact representation (latent variable) that maintains sufficient information for a given predictive task [76,77,78].

On the other hand, the formal relationship of the IB problem with the error exponents of the distributed test against independence can be explicitly seen in the proof presented by Espinosa et al. in Ref. [15] (Equation (38)). More precisely, they analyzed the discrepancy between the fundamental limit (the single-letter expression of $P_{X,Y}$) given in Equation (48) and its corresponding multi-letter operational expansion, i.e., the following expression:

(49) $$\max_{\begin{array}{c} U:U\;―\;○\;X\;―\;○\;Y\\ I(U;X)\leq R\;|U|\leq |X|+1 \end{array}}I\left(U;Y\right)-\max_{{\tilde{f}}_{l}:X^{l}\to \left\{1,\ldots ,2^{lR}\right\}}\frac{1}{l}I\left({\tilde{f}}_{l}\left(X_{1}^{l}\right);Y_{1}^{l}\right),$$

The authors in Ref. [15] called this the non-asymptotic analysis of the *information bottleneck (IB) problem*. Finally, modern machine learning methods can be used to learn (from the data) the encoders and the decoders needed in distributed HT. This approach has been explored by Espinosa et al. [79].

To summarize, the importance of the IB problem in machine learning lies in its ability to provide a practical framework to design an encoder–decoder scheme, addressing the exciting dimension of lossy compression for generalization in learning. In contrast, in the distributed HT setup presented in Section 6 of this survey, the IB optimization is the fundamental performance limit (describing the error exponent of the task), i.e., the IB problem represents the complexity of this distributed inference task. Therefore, the IB problem provides a natural bridge between distributed HT, which is the focus of this survey, and learning algorithms with an encoder–decoder structure, which is very popular nowadays in representation learning.

### 8.2. Error Exponents in Communication Networks

This subsection introduces the application of error exponents in current communication systems, highlighting how they can improve performance or reduce resource usage. Systems with certain characteristics, such as high-stakes decision-making and the need for reliable verification, are prime candidates for using error exponents. These systems benefit from the error exponent analysis’s enhanced accuracy and efficiency. In the following subsections, we will discuss different communication architectures and how the application of error exponents impacts their performance and resource usage.

#### 8.2.1. Vehicular Networks

A vehicular network (VN) uses the location reported by neighboring vehicles to make decisions in real time, e.g., the coordination of crossings in an automated intersection, a lane-changing application, etc. If a vehicle fakes its reported location, it creates chaos in the decision-making process that may even be deadly. Ref. [80] deals with a VN where location information is critical for various safety and operational applications. This includes applications such as the coordination of crossings in automated intersections and lane-changing systems. The main hypothesis is that the reported location of vehicles can be erroneous or maliciously falsified, which can disrupt the network’s decision-making processes and potentially lead to dangerous situations. The authors utilize HT to design a location verification system (LVS) to handle unknown channel conditions and threat models. In particular, they define $H_{0}$ as the hypothesis that a vehicle’s reported location is accurate and $H_{1}$ as the hypothesis that it is falsified. Then, they use the collected signal metrics to calculate the likelihood ratio for each hypothesis and derive the probability of errors using the Chernoff bound or the large deviation principle. In this HT context, error exponents can be used as a proxy to calculate the probability of error due to the high computational cost of evaluating the full analytical expression of the probability of error [81]. This approach simplifies the process, making it more feasible to implement in vehicular systems where computational resources and time are limited.

#### 8.2.2. Wireless Sensors

In Ref. [16], the authors provide a classical decentralized detection framework, where each sensor node in a network independently processes its observations before transmitting a summary to a central fusion center. This network has limitations due to resource constraints, such as cost, bandwidth, and power. The authors propose an alternative theoretical framework tailored to modern sensor networks, emphasizing the optimization of sensor node designs and fusion rules. Key findings include the optimality of using identical local decision rules under conditional independence assumptions and the application of error exponents to assess system performance. Remarkably, error exponents play a critical role in this work as they provide a quantitative measure of how quickly the probability of error decreases as the number of sensors or the resource budget increases. The authors can decouple the optimization problem across sensors by focusing on the error exponents, allowing for more straightforward and effective design strategies. This metric helps compare different system configurations and ensures that the detection performance improves asymptotically as the number of sensors increases.

#### 8.2.3. Unordered Data in Sensor Network

Another concrete application of error exponents can be found in Ref. [24]. The authors address the classical problem of testing two simple statistical hypotheses with the twist that the data vector is observed after an unknown permutation of its entries. The authors explore the fundamental limits of detection performance under this scenario, quantifying how much information is contained in the values of the entries versus their positions. The first part of the paper answers these theoretical questions while the second part focuses on practical algorithms for detection without estimating the permutation. In this performance analysis, error exponents play a crucial role in characterizing the performance limits of detection tests with unlabeled data. The authors introduce the concept of the error exponent for unlabeled detection, quantifying the rate at which the probability of type II error decreases exponentially as a function of the data size *n*. They demonstrate that for any fixed rate of decrease in a Type I error, the Type II error cannot decrease faster than the error exponent, and it is bounded between the error exponents for labeled data. This framework allows for comparing the efficiency of different detection algorithms under the constraint of unknown data permutations. Moreover, the authors in Ref. [24] developed several algorithms focusing on theoretical (error exponents) limits and practical implementation. These tests attempt to estimate the unknown data permutation under each hypothesis. The practical implications of these algorithms are significant, especially in applications where data labeling is either impossible or impractical. Some key points include large sensor networks and individual sensor identities that might be unknown or unreliable due to communication constraints. The proposed algorithms enable robust HT even without knowing which sensor provided which data point.

### 8.3. Error Probability Estimation in Vehicular Networks

As seen in Ref. [16], using error exponents can significantly enhance the system’s ability to estimate the probability of error. Given the high computational cost of evaluating the full analytical expressions for error probabilities, error exponents serve as a practical proxy by providing an exponential measure of how quickly these probabilities decay as more information is gathered. By integrating error exponents into the location verification system, the vehicular network can replace the traditional probability of error bounds with error exponent approximations. This allows for the system to be arbitrarily close to the asymptotic limit as the number of samples increases. Consequently, this approach reduces the computational overhead and ensures accurate and reliable verification, which is essential for real-time decision-making in dynamic vehicular environments. This method optimizes resource use while maintaining the high levels of safety and performance required in such critical applications.

### 8.4. Numerical Analysis in Distributed HT

To conclude this section, we conduct a simple numerical analysis to illustrate the potential use of some of the error exponents presented in this survey. This numerical analysis follows the design, interpretations, and derivations introduced in Ref. [15]. We will focus on the emblematic case of the test against independence to illustrate the adoption of Theorem 8 to bound $\beta_{n}\left(\epsilon_{n},R\right)$ with finite-sample size *n*. This analysis follows from both the non-asymptotic expressions presented in Theorem 8. These expressions allow us to determine the sample size needed to closely approximate finite-length performance ($\beta_{n}\left(\epsilon_{n},R\right)$) with its respective error exponent approximation: exp(−nξ(R)). Importantly, this error exponent proxy can be computed numerically. In concrete terms, Theorem 8 offers an interval of feasibility for $\beta_{n}\left(\epsilon_{n},R\right)$ expressed by $\beta_{n}\left(\epsilon_{n},R\right)\in \left[\mathrm{LB}\left(\epsilon_{n},R\right),\mathrm{UB}\left(\epsilon_{n},R\right)\right]$ where $\mathrm{LB}(\epsilon_{n},R)$ corresponds to Equations (32), (34), (36), and (38) and $\mathrm{UB}(\epsilon_{n},R)$ corresponds to Equations (33), (35), (37), and (39) for the different regimes of ${\left(\epsilon_{n}\right)}_{n}$.

**Figure 4:**
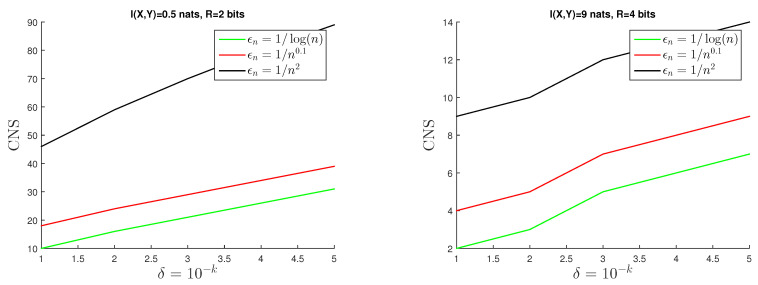
CNSs predicted by Theorem 8 across different values of $\delta =10^{-k}$. The values used are for I(X;Y)=0.5, with R=2 for the low-rate case and I(X;Y)=9, R=4 for the high-rate case.

The authors in Ref. [15] claim that the expression exp(−nξ(R)) can be adopted as a practical proxy to $\beta_{n}\left(\epsilon_{n},R\right)$. To support this claim, for a given small δ>0 of the form $10^{-k}$ with $k\in \left\{1,..,5\right\}$ and a joint model $P_{XY}$, they find the lowest *n* such that $\beta_{n}\left(\epsilon_{n},R\right)\in \left(e^{-n\xi (R)}-\delta ,e^{-n\xi (R)}+\delta \right)$. The exponential decay of the length of the interval $\left[\mathrm{LB}\left(\epsilon_{n},R\right),\mathrm{UB}\left(\epsilon_{n},R\right)\right]$ with *n* (from the expressions in Theorem 8) suggests that this condition happens with *n* very quickly. Importantly, the authors in [15] derive an upper bound for this Critical Number of Samples (CNSs) from the closed-form expressions derived for $\mathrm{LB}(\epsilon_{n},R)$ and $\mathrm{UB}(\epsilon_{n},R)$—the predicted CNSs is the first n≥1 such that $\max\{\mathrm{UB}\left(\epsilon_{n},R\right)-e^{-n\xi (R)},e^{-n\xi (R)}-\mathrm{LB}\left(\epsilon_{n},R\right)\}\leq \delta$, which is finite for any δ>0 and can be computed from Theorem 8. Following the same experimental setting and analysis proposed and presented in Ref. [15], Section V, Figure 4 illustrate the predicted (from Theorem 8) CNSs vs. $\delta =10^{-k}$ for different scenarios of $P_{XY}$ (in terms of the magnitude of I(X;Y)) and ${\left(\epsilon_{n}\right)}_{n}$. We use a discretized version of a Gaussian pdf $P_{XY}$ of |X|×|Y| where the mutual information between the two variables (*X* and *Y*) is 0.5 and 9 nats, respectively, and we explore ${\left(\epsilon_{n}\right)}_{n}\in \left\{n^{-2},n^{-0.1},1/\log\left(n\right)\right\}$. Figure 4 show that even for a very small precision $\delta =10^{-5}$, the point at which $\beta_{n}\left(\epsilon_{n},R\right)$ is approximated by $e^{-n\xi (R)}$ occurs with fewer than 14 samples for the high-rate case and in fewer than 90 samples for the low-rate case for the majority of ${\left(\epsilon_{n}\right)}_{n}$. The dependency of these CNS values on the magnitude of I(X;Y) and ${\left(\epsilon_{n}\right)}_{n}$ is clearly expressed.

A more complete analysis and discussion of these numerical approximations can be found in [15].

## 9. Final Discussion

This survey explores the interpretations and significance of the information-theoretic error exponent analysis of hypothesis testing. It starts with the standard centralized case and then addresses the challenges introduced by distributed scenarios with multiple agents and communication constraints. We cover many results in the literature that explain the sensitive effect of communication restriction (limitation) in performance in the form of asymptotic and non-asymptotic results. We show the importance of deriving fundamental information limits (error exponents) and their relevance in a finite-length (non-asymptotic) performance analysis. On this, error exponents offer interesting interpretations and have the power to inform the design of practical schemes operating in realistic (non-asymptotic) conditions. Table 1 provides a big-picture overview of the presented results.

This survey underscores the crucial link between error exponents and error probabilities. Understanding this connection is paramount in devising effective strategies for HT, as it provides insights into the inherent trade-offs between communication efficiency and inference performance and informs the ways to design (from data) practical encoders and decoders [79]. By leveraging insights gleaned from error exponents, researchers and practitioners could better understand the complexities of HT in distributed environments and gain insights and knowledge to advance the frontier of statistical inference.

### Open Problems and Further Work

The results covered in this survey point out a few promising areas for future research. One important technical topic is addressing the problem of arbitrary binary hypothesis testing (HT) under communication constraints, as discussed in Section 4. A single-letter characterization of the Type II error exponent is yet to be determined, with only a lower bound derived in [60]. It would be a significant achievement to characterize this fundamental limit.

Another relevant area to consider is expanding the collaborative findings presented in this survey to situations with finite-length sample sizes and scenarios involving multiple rounds of node interactions. This is a complex task because determining an error exponent for the Type II error over many rounds is not a simple extension of the results discussed in this survey.

## Figures and Tables

**Table 1:** A summary of the main results for all the different contexts of hypothesis testing communication settings.

	BHT	DHT (R≥H(X))	DHT (R<H(X))	DHT: Against Independence	Interactive HT
EE (ϵ>0 fixed)	D(P||Q)	$D(P_{X,Y}||Q_{X,Y})$	Theorems 4 and 5	Theorem 6	Theorem 9
EE ($\epsilon_{n}\to 0$)	D(P||Q)	$D(P_{X,Y}||Q_{X,Y})$	Theorems 4 and 5	Theorem 7	Theorem 9
EE ($\epsilon_{n}=e^{-nr}\to 0$ )	Theorem 1	Theorem 1	[49]	Proposition 1	-
FL Bounds (ϵ>0)	Theorem 2	Theorem 2	-	Theorem 8	-
FL Bounds ($\epsilon_{n}\to 0$)	Theorem 3	Theorem 3	-	Theorem 8	-

## Appendix A

Rate-distortion theory [1,73] refers to coding theorems in information theory that deal with data compression subject to a fidelity constraint. The *rate-distortion function*, R(D), characterizes the minimum rate *R* at which data can be encoded such that the distortion *D* between the original and reconstructed source does not exceed a specified threshold. Conversely, the *distortion-rate function*, D(R), defines the minimum distortion achievable at a given encoding rate *R*. These functions are crucial in applications such as lossy data compression, where one seeks to minimize the amount of data resources while preserving an acceptable level of quality. The rate-distortion function is mathematically expressed as

$$R\left(D\right)=\underset{P_{\tilde{X}|X}:E\left[d\left(X,\tilde{X}\right)\right]\leq D}{\inf}I\left(X;\tilde{X}\right),$$

where $I(X;\tilde{X})$ denotes the mutual information between the source *X* and its reconstruction $\tilde{X}$, and $d(X,\tilde{X})$ is a distortion measure. On the other hand, the distortion-rate function is

$$D\left(R\right)=\underset{P_{\tilde{X}|X}:I\left(X;\tilde{X}\right)\leq R}{\inf}E\left[d\left(X,\tilde{X}\right)\right],$$

where $E[d\left(X,\tilde{X}\right)]$ is the expected distortion between the source and the reconstruction given that the mutual information between them does not exceed the rate *R*.
